# Easiness of Use and Validity Testing of *VS-SENSE* Device for Detection of Abnormal Vaginal Flora and Bacterial Vaginosis

**DOI:** 10.1155/2010/504972

**Published:** 2010-10-07

**Authors:** Gilbert G. G. Donders, Camila Marconi, Gert Bellen

**Affiliations:** ^1^Femicare Clinical Research for Women, Lombardstraat 28, 3300 Tienen, Belgium; ^2^Department of Obstetrics and Gynaecology, Regional Hospital H Hart, Tienen, Belgium; ^3^University Hospital Gasthuisberg, Leuven, Belgium; ^4^Department of Pathology, Botucatu Medical School, São Paulo State University, Rubiao Jr. District, 18618-970 Botucatu, SP, Brazil

## Abstract

Accessing vaginal pH is fundamental during gynaecological visit for the detection of abnormal vaginal flora (AVF), but use of pH strips may be time-consuming and difficult to interpret. The aim of this study was to evaluate the *VS-SENSE* test (Common Sense Ltd, Caesarea, Israel) as a tool for the diagnosis of AVF and its correlation with abnormal pH and bacterial vaginosis (BV). The study population consisted of 45 women with vaginal pH ≥ 4.5 and 45 women with normal pH. Vaginal samples were evaluated by *VS-SENSE* test, microscopy and microbiologic cultures. Comparing with pH strips results, *VS-SENSE* test specificity was 97.8% and sensitivity of 91%. All severe cases of BV and aerobic vaginitis (AV) were detected by the test. Only one case with normal pH had an unclear result. Concluding, *VS-SENSE* test is easy to perform, and it correlates with increased pH, AVF, and the severe cases of BV and AV.

## 1. Introduction

Abnormal vaginal discharge is one of the most frequent complaints in women attending gynaecological clinics. Frequently abnormal discharge is related to abnormal vaginal flora (AVF), such as bacterial vaginosis (BV), trichomoniasis, and aerobic vaginitis (AV) [1–4]. Despite the unpleasant sensation reported by the women suffering of vaginal infections, these infections have been correlated with adverse pregnancy outcome [5–7], as well as with increased risk of sexually transmitted diseases such as HIV [8].

Increased pH is found in vaginal samples of most women with AVF. Determining vaginal pH is a useful screening tool for the diagnosis of AVF, but this procedure may be time consuming during gynaecological examination. Additionally, most practitioners measure vaginal pH using pH indicator strips, whose colour scale can be difficult to interpret [9]. Therefore the utilization of a fast and user-friendly test to detect abnormal pH indicative of AVF would be useful and could contribute for less doubtful results.

The *VS-SENSE* (Commom Sense Ltd, Caesarea, Israel) consists in a vaginal swab, which includes the nitrazine yellow pH indicator. It was developed aiming to detect abnormalities in the pH values and in the buffer capacity of the vaginal secretion, which can be indicative of vaginal infections, such as BV and trichomoniasis (see website http://www.cs-commonsense.com/content.asp?pageid=93). In the presence of vaginal secretion with abnormal parameters, the swab colour changes resulting in a positive test. The initially yellow-coloured swab may turn into green or blue in the presence of abnormal pH (>4.7) and any slight change in the swab colour should be considered a positive result. According to the manufacturer, and it can be used by physicians during consultation or even by patients presenting abnormal vaginal discharge. 

Therefore, the aim of this study was to evaluate the usefulness of the *VS-SENSE* test to detect an abnormal pH and as an additional tool for the physician or, by extension, even for the self-diagnosis by the patient herself of abnormal vaginal flora, as well as evaluate the correlation of this test with presence of bacterial vaginosis.

## 2. Material and Methods

### 2.1. Study Population and Inclusion Criteria

From September until November 2009, 90 women attending the vulvovaginitis clinic at Gasthuisberg University Hospital, Leuven, Belgium, were included in the study. During the gynaecological examination, vaginal pH was routinely measured, microscopy of fresh vaginal fluid was performed, and vaginal cultures were taken routinely in all women attending the clinic. Subjects were included in the *VS-SENSE * accuracy study when the vaginal pH was equal or superior to 4.5, while the next following patient whose pH was normal (between 3.6 and 4.4) was included as a control patient. Cut-off of pH = 4.5 was established based on one of the criteria for the BV diagnosis according to Amsel et al. [10]. If the next patient also had an abnormal pH, the following 2 patients with normal pH were included as controls. Case and control groups were composed of 45 women each. Exclusion criteria were age below 18, pregnancy, menopausal status, menstruation, vaginal douching, treatment for vaginal infections in the previous 14 days, and sexual intercourse in the previous 12 hours.

### 2.2. Samples Collection

All samples were collected by a physician during gynaecological examination. With two fingers spreading the vulva, a polyethylene swab was gently inserted in the introitus and pushed up to the upper vault of the vagina. The swab was pressed on a pH strip (Macherey-Nagel GmbH & Co, Duren, Germany) for 5 seconds and the material of the swab was spread on a glass for the microscopic evaluation. After this, the swab was placed on Amies transport medium for accessing the microbiologic culture profiles. The *VS-SENSE* test swabs were introduced in the vagina in a similar way allowing contact with vaginal content for 10 seconds. After removal from the vagina, the swabs were observed by 3 persons for determination of the final colour. According to the manufacturer's instructions, the originally light-yellow swab changes to green or blue after contact with samples with bacterial vaginosis, or it remains unchanged with yellow coloration in case of normal microflora. The colour of the swab was based on the concordant opinion of at least 2 observers. If all 3 observers agreed with the result, swab colour was defined as clear, if not, colour reading was defined as unclear. The first observer was aware if the sample belonged to case or control group while the 2 other observers were blinded. After analysing the swab colour, the first observer showed the sample to the other observers individually who communicate their opinion independently. Thus, the first observer compares the opinions and classifies them as clear or unclear.

### 2.3. Microscopic Assessment of the Vaginal Microflora

Immediately after collection of the smears, one droplet of saline was added to the glass and microscopic evaluation was performed in ×400 magnification in a phase-contrast microscope (Leica MD1000, Wetzlar, Germany). The protocol for classification of AVF, aerobic vaginitis, full or partial bacterial vaginosis was done according toDonders[3].Lactobacillary grades (LBG) were classified as LBG I when *Lactobacillus *morphotypes were predominant, LBG IIa as a normal lactobacillary pattern but with significant quantity of non-*Lactobacillus* morphotypes, LBG IIb when non-lactobacillary flora exceeded the quantity of *Lactobacillus *and finally the LBG III when the total replacement of the lactobacillary flora by other morphotypes was observed. Diagnosis of bacterial vaginosis was performed according to the extension of the typical anaerobe granular microflora in the sample. If the typical granular flora of BV-like bacteria was observed all over the smear or if there were more than 20% of epithelial cell covered with bacteria (clue-cells) it was diagnosed as “Full BV." When less than 20% of clue cells were observed or there were areas with streaks of BV-like bacteria mixed with other morphotypes, it was classified as “Partial BV" [6]. Aerobic vaginitis flora was defined as the presence of small bacilli and/or cocci in pairs or chains and scored according to the severity, taking into account the lactobacillary grade, pattern of the background, number of leucocytes, presence of toxic leucocytes, and presence of parabasal cells [4]. Candidiasis was diagnosed when unequivocal pseudohyphae and/or blastophores were detected on the smears and/or microbiology cultures resulted positive for *Candida* sp. Vaginal trichomoniasis was diagnosed if parasites with morphology and motility characteristic of *Trichomonas vaginalis *were visualized in the wet mount smears in the microscopic analysis. PCR for *T. vaginalis *was performed only when wet mount evaluation was inconclusive on its presence. *T. vaginalis* is infrequent in this population, its prevalence being 0.66% [11].

### 2.4. Microbiologic Cultures

The cotton swab from the vaginal wall was collected and transported to the microbiology laboratory in the Amies medium. Bacterial and yeast culture were performed in all samples and the microorganisms were semiquantified from 1+ to 3+, according to the number of yielded colonies.

### 2.5. Clinical Data

Oral consents were obtained from all patients and all relevant clinical data and medical history information were taken by filing a form specifically designed for the study. Information concerning the gynaecological history, days of last sexual intercourse, previous vaginal infections, and presence of current symptoms and discharge were collected. After informing about the need for IRB approval, we were told this was not necessary as the test was just a double check on a pH test that was done already routinely on every patient, comparable to testing a new device or colouring technique on samples in a chemical or pathology lab.

### 2.6. Statistical Analysis

Chi² of Fisher exact were used as appropriate for categorical variables. Student's is used for normally distributed continuous variables like pH values and age, or Welch's equation if not normally distributed.

## 3. Results

In the period from September 2008 to March 2009, 227 women were seen at the vulvovaginitis clinic at Gasthuisberg University Hospital, Leuven, Belgium. Abnormal vaginal pH were found in 63 (27.8%) of the patients. The evaluation with *VS-SENSE *test swabs were performed in vaginal samples from 45 women with pH ≥ 4.5 and from the subsequent 45 women with normal pH fitting the inclusion/exclusion criteria. Although additional 18 women presented abnormal pH, their samples were not assessed by *VS-SENSE test* swabs by the presence of blood in the samples, recent intercourse, pregnancy or by their menopausal status (exclusion criteria).

Demographics and gynaecological data are presented in Table 1. Patients from the study group, and therefore presenting abnormal pH had significant higher rates of previous episodes of BV and AV, and borderline lower rates of previous acute or chronic candidiasis.

As in the control group (pH < 4.5) only 1 false-positive case was detected, specificity of the test was as high as 97.8%. In the case group (pH ≥ 4.5), there were approximately 9.0% false negative cases accounting for a sensitivity of 91% (Table 2). There was divergence of opinion in between the 3 observers 18 (20.0%) samples, thus considered unclear results. Unclear colour was observed only rarely in normal controls (1 case), while in the women with pH of 4.5 or above, green and blue swabs were considered unclear in approximately 1/3 and 2/3 of the cases, respectively (Table 3). Swabs presenting green colour had the highest percentage of unclear readings, with a disagreement among observers of over 40% of the cases (10/22 unclear green versus 4/52 unclear yellow, *P* = .0005), followed by blue readings (4/16 unclear blue versus 4/52 unclear yellow, *P* = .08). 

The comparison between *VS-SENSE *test and microscopy result of the vaginal smears is shown in Table 4. From the total of 40 samples with normal flora, 3 (7.5%) had positive swab results. In 15 (30%) cases of AVF, the swab colour remained yellow indicating a false negative test. All 4 cases with severe AV were detected by *VS-SENSE*, but from the 23 samples presenting the diagnosis of mild AV, only 14 (60.0%) had positive swab results. Similarly, all 11 cases with microscopic diagnosis of full BV were detected by the change of the swab colour, while only one of the 3 cases with partial BV was detected by the swab test. Twelve of 14 (85.7%) women with candidiasis (by microscopy and/or microbiologic culture) had a negative result by the swab colour evaluation. All positive samples for candidiasis, but with negative result in the swab test had also normal LBG by microscopy (data not shown).

The microscopic evaluation allowed the identification of 2 cases of normal vaginal flora in the case group (pH ≥ 4.5) which were also positive by *VS-SENSE *test (Table 5). Among the samples with normal pH and negative *VS-SENSE *test, one case of LBG III was detected. In the control group, one case with pH of 3.8 and normal microscopy result revealed a blue final colour in the swab test. Blue colour was a stronger indicator of AVF (LBG III) than green, which by its turn reflects better abnormal results than yellow.

The microbiologic culture results showed overgrowth of bacteria in approximately 19% of the samples (Table 6). Among the culture-positive cases, 4 presented normal results by microscopy, all of which were carrying gram-positive Group-B streptococci (GBS) only. The remaining 13 had samples, had AVF on microscopic evaluation, and showed overgrowth of the gram-negative *Escherichia coli *and *Klebsiella pneumoniae*, in addition to GBS. Thirteen of 17 culture-positive samples also showed positive *VS-SENSE *test (76.5%).

## 4. Discussion

Our findings revealed that *VS-SENSE *test is a highly specific test for detection of normal pH and vaginal flora, and can hence be considered an important tool for the reassurance of normal vaginal conditions. Furthermore, in normal women the yellow colour can easily be recognized, underlining its user-friendliness. Therefore, *VS-SENSE *test can be considered an easy to perform and very good test to exclude abnormal pH and consequently AVF. 

The difficulty in the recognition of the swab colours was already reported by the group of Sobel et al., although their rate of doubtful cases was inferior to ours [12, 13]. This may be due to the evaluation method adopted in the present study, in which the agreement of 3 observers was needed in order to call a test result unequivocally clear. Considering that green and blue colours had the highest rates of disagreement, differentiation between these 2 colours might not be relevant if both would equally represent abnormalities in pH and AVF. According to the manufacturer, any change in the swab colour should be considered as positive. Although numbers were small, our results did not seem to indicate that abnormal pH, abnormal vaginal flora and bacterial vaginosis were more frequent in blue than in green test results. But as green colours were twice as frequent unclear than blue colours, it must be decided whether they are in such cases truly positive or not. Our data clearly indicate that most of the doubtful green and blue results were in fact found in women with abnormal flora and/or high pH, indicating that in case of doubt, a slight discoloration from yellow to green or blue is to be considered positive. It is not known whether retesting shows less equivocal results. 

Another issue is high rate of false-negative findings with the *VS-SENSE *test, according to our findings as high as around 30% of cases with AVF that would be missed if only performing the swab test. Since we included both LBG 0 and IIb in the definition of AFV in this paper, this wide definition could most likely contribute to a high false-negative rate. Detection and management of intermediately disturbed flora has caused much controversy in the literature [3, 14]. Several studies show that intermediate flora relates to bad pregnancy outcome [6, 7, 15, 16]. For this reason, the use of *VS-SENSE *to detect high-risk flora types during pregnancy should at the present time not be encouraged. 

Concerning detection of more specific diseases, all severe cases of AV were properly detected by the *VS-SENSE *swab, while 60% of the mild cases were found. Also all cases of full BV were detected, but less so the cases with partial BV. So despite the *VS-SENSE *had a significant rate of false-negative cases for the detection of AVF, it was an efficient method to detect the most severe abnormalities of AV and BV. As could be expected, due to the absence of a link with abnormal flora or pH, we provided confirmative evidence that *VS-SENSE *test is of no use in the detection of vaginal candidosis, nor colonisation with Candida. However, the statement of the manufacturer, that cases of vaginal candidosis should result in yellow swab colour, is not justified either, as this is only observed if also the LBG is normal. Due to the absence of cases with trichomoniasis in this series, the test value for this condition could not be tested.

Finally, given the 10% false-negative test results of the test system compared to increased pH value, however, one can discuss the necessity to repeat testing or additional tests in doubtful cases or when complaints are present. The data regarding the pH values and LBG found in each swab colour group shows that blue swabs correlate better with abnormal pH and AVF, when compared to green. The 2 cases with increased pH and *VS-SENSE *test positive, but classified as LBG I by microscopy, may partially be explained by recent sexual intercourse (24 hours), as found in one of the cases or by other influences on the pH without disturbing the bacterial flora. The *VS-SENSE TEST *is not advised for use in the event of recent sexual intercourse. The one sample with normal pH and negative swab test result in the swab test, but diagnosed as LBGIII and mild AV flora may be explained by the presence of coarse morphotypes of lactobacilli, such as the recently recognized *Lactobacillus iners* [17], that can be misinterpreted as astaphoidflora [18].

It was rather surprising that also aerobic microbiologic cultures results were associated with abnormal swab results. Not only pH or microscopy, known as likely indicators of an abnormal test, but also overgrowth of *E. coli *and *K. pneumoniae* were linked to a positive test. The clinical meaning of positive cultures on vaginal samples, however, is being a matter of discussion, since presence of positive culture is not a reason for treatment, unless in the presence of abnormal microscopy results and concordance with complaints. 

We realize the positive predictive value of the test could be different in a random (screening) setting, as the lower prevalence of abnormal pH (e.g., 5% to 10%) underlines the importance of a high specificity. In this study, we choose for the scenario of cases versus controls, because it resembles better the real clinical setting. Indeed, we assume the test will mostly be used to test symptomatic women, not to screen asymptomatic women. Whereas specificity is more important in a setting with low prevalence (prevent unnecessary interventions), sensitivity should be high in a setting with high prevalence (not to miss to many cases).

It may need some explanation why it was chosen to use fresh wet mounts for AVF and BV testing and not the Gram stained Nugent's score. Wet mount reading allows better validation of lactobacillary flora grades than Gram stains, probably because of some lactobacilli get lost during the Gram fixation and coloring process, leading to a false increase in abnormal lactobacillary grades [19]. Furthermore, it allows better distinction between different flora types like BV, partial BV, and AV [4]. Finally, the study design using wet mounts correlates more to the everyday's practice—or at least what it should be—where sending of a specimen for Nugent is impractical due to many reasons [3].

Other points of care tests are available for the diagnosis of BV and abnormal pH. For example, the BVBlue test has been shown to be sensitive and specific [20], but their results are not directly comparable to ours. As discussed above, we envisioned not only to detect BV, but also other types of abnormal vaginal flora. For the VS Sense device tested in this study, a negative test excludes well the presence of disease, but some cases with AVF can still be missed.

In summary, we provide evidence that the *VS-SENSE *is an easy to perform test and it correlates well with increased pH and AVF, as well with severe cases of BV and AV infection. However, false negative results may occur and negative tests in the presence of clinical suspicion should prompt for retesting or additional testing or advice. Discerning between green and blue tests is not useful, but of both colours being more often unclear to read, the former has very often doubtful readings. According to our data, these doubtful cases should usually be seen as true positives.

## Figures and Tables

**Table 1:** Demographic and gynaecological characteristics of the women included in the study, divided into case and control groups, composed by 45 women each.

Characteristics	Case *n* (%)	Control *n* (%)	*P* value
Age*^a^	37.2 ± 11.9	35.1 ± 9.9	*P* = .56

Parity^b^			
Nulilparous	26/43 (60.8)	30/44 (68.2)	*P* = .60
Multiparous	17/43 (39.5)	14/44 (31.8)	*P* = .60

History^b^			
Candida	19 (42.2)	29 (64.4)	*P* = .057
Recurrent candida	10 (22.2)	19 (42.2)	*P* = .07
BV	11 (24.2)	1 (2.2)	*P* = .005
AV	17 (37.8)	2 (4.4)	*P* = .0003

pH*^a^	5.3 ± 0.4	3.9 ± 0.3	*P* < .0001

Symptoms/complains^b^			
Burning	13 (28.9)	12 (26.7)	*P* = 1.0
Itching	10 (22.2)	10 (22.2)	*P* = .8
Pain	15 (33.3)	16 (35.5)	*P* = 1.0
Discharge	21 (46.7)	10 (22.2)	*P* = .026

*Data expressed in mean ± sd.

^a^Student's test (normal distribution) or Withney Rank Sum test (not normal distribution).

^b^Chi^2^ or Fisher exact test.

**Table 2:** Sensitivity and specificity of *VS-SENSE *test with pH less than 4.5 or more as discrimatie factors. Sensitivity: 82.2%, Specificity: 97.8%, Positive predictive value: 97.4%, and Negative predictive value: 84.6%.

	Case group (pH ≥ 4.5)	Control group (pH < 4.5)	Total
Positive	37 (82.2)	1 (2.2)	38
Negative	8 (17.8)	44 (97.8)	52

Total	45	45	90

**Table 3:** Results of *VS-SENSE *test and colour classification in clear and unclear, according to the agreement among observers.

	Clear *n* (%)	Unclear *n* (%)	Total (%)
Case group (pH ≥ 4.5)	28 (62.2)	17 (37.8)***	45 (100.0)
Yellow	5 (11.1)	3 (6.7)	8 (17.8)
Green	12 (26.7)	10 (22.2)	22 (48.9)
Blue	11 (24.4)	4 (8.9)	15 (33.3)

Control group (pH < 4.5)	44 (97.8)	1 (2.2)	45 (100.0)
Yellow	43 (95.6)	1 (2.2)	44 (97.8)
Green	0	0	0
Blue	1 (2.2)	0	1 (2.2)

Total	72 (80.0)	18 (20.0)	90 (100.0)

***P < .0001 versus control group. Fisher exact test.

**Table 4:** Results of *VS-SENSE* test showing the number of cases according to pattern of vaginal flora presented by microscopic evaluation, *Candida* infection and vaginal pH value.

	Negative	Positive	Total
Normal^a^	37 (41.1)	3 (3.3)	40 (44.4)
Abnormal^b^	15 (16.7)	35 (38.9)	50 (55.6)

AV			
Mild	9 (10.0)	14 (15.6)	23 (25.6)
Moderate/severe	0	4 (4.4)	4 (4.4)

BV			
Partial	2 (2.2)	1 (1.1)	3 (3.3)
Full	0	11 (12.2)	11 (12.2)

AV + BV	4 (4.4)	2 (2.2)	6 (6.6)
Candida^c^	12 (13.3)	2 (2.2)	14 (15.5)

pH			
<4.0	44 (48.9)	1 (1.1)	45 (50.0)
≥4.5	8 (8.9)	37 (41.1)	45 (50.0)

^a^Lactobacillary grade (LBG) I or IIa in microscopic evaluation; ^b^LBG 0, IIb, or III in microscopic evaluation; ^c^Presence of positive culture for* Candida *sp. or detection of sporae or hyphae by microscopy.

**Table 5:** *VS-SENSE *test and vaginal pH values according to the lactobacillary grade (LBG) determined by microscopic evaluation of fresh vaginal smears. Differences according to Kruscal-wallis test.

	Case (*n* = 45)						Control (*n* = 45)			
	Yellow		Green		Blue		Yellow		Blue	
LBG	*n* (%)	pH*	*n* (%)	pH* *n* (%)	pH*	*n* (%)	pH*	*n* (%)	pH*
0	0		2 (9.1)	5,5 ± 0,2	1 (6.7)	5,6	0		0	
I	0		1 (4.6)	4,8	1(6.7)	5,3	35 (79,5)	3,9 ± 0,3	1 (100.0)	3,8
lIa	0		0		0		2 (4,6)	4,1	0	
Ilb	4 (50,0)	5,0 ± 0,3	5 (22,7)	5,3 ± 0,5	0		6 (13,6)	4,2 ± 0,3	0	
Ill	4 (50,0)	4,8 ± 0,3	14 (63,6)	5,2 ± 0,4	13 (86.6)	5,6 ± 0,4	1 (2,3)	4,2	0	
Total	8 (17,8)	5,0 ± 0,4^a^	22 (48,9)	5,2 ± 0,4^b^	15 (33,3)	5,6 ± 0,4^c^	44 (97,8)	3,9 ± 0,3	1 (2,2)	3,8

*pH values expressed in mean ± sd.

a versus b: *P* = .234.

b versus c: *P* = .005.

a versus c: *P* = .004.

**Table 6:** Results of microbiologic cultures of vaginal samples from cases and controls with normal and abnormal microscopic evaluation, according to the *VS-SENSE* test result.

Microscopy	Positive culture^a^	Negative culture	Total
	*n* (%)	*n* (%)	*n* (%)
Normal	4 (4,4)^b^	36 (40,0)	40 (44,4)
Yellow swab	3 (3,3)	34 (37,8)	37 (41,1)
Green or blue swab	1 (1,1)	2 (2,2)	3 (3,3)

Abnormal	13 (14,4)^c^	37 (41,2)	50 (55,6)
Yellow swab	1 (1,1)	14 (15,6)	15 (16,7)
Green or blue swab	12 (13,3)	23 (25,6)	35 (38,9)

Total	17 (18,8)	73 (81,2)	90 (100)

^a^Positive cultures defined as 2 or 3+ of yielded colonies. ^b^Species identified: Group-B streptococci (GBS). ^c^Species identified: GBS, *Escherichia coli*, *Klebsiella pneumoniae*.
